# A single-institutional retrospective analysis of factors related to vaginal intraepithelial neoplasia

**DOI:** 10.1186/s12905-023-02714-4

**Published:** 2023-10-24

**Authors:** Hongmin Zeng, Qianling Dai, Dan Jiang

**Affiliations:** Chengdu Women’s and Children’s Central Hospital, No. 1617, Riyue Avenue, Qingyang District, Chengdu, Sichuan Province China

**Keywords:** Vaginal intraepithelial neoplasia, Cervical intraepithelial neoplasia, Risk factors

## Abstract

**Background:**

To date, few studies on the factors related to vaginal intraepithelial neoplasia (VaIN) have been published. In this study, we aimed to analyze the features of VaIN and identify underlying risk factors.

**Methods:**

Patients with VaIN or vaginitis histologically confirmed at the Industrial Street Branch of Chengdu Women’s and Children’s Central Hospital from July 2020 to December 2021 were included. We statistically analyzed their baseline clinical characteristics, human papillomavirus (HPV) infection status, cytology results, and pathology results. Categorical indicators were analyzed using the chi-square test or Fisher’s exact test, as appropriate. Differences were considered to be statistically different with p < 0.05.

**Results:**

A total of 62 patients with VaIN (mean age: 39.06 ± 11.66 years) and 32 with vaginitis (mean age: 41.13 ± 13.43 years) were included. Synchronous cervical intraepithelial neoplasia (CIN) was histologically identified in 46 (74.2%) patients with VaIN and 7 (21.9%) with vaginitis (p < 0.001). Low-grade squamous intraepithelial lesions (LSILs) and atypical squamous cells of undetermined significance (ASC-US) were the most frequent cytological abnormalities in both groups. Patients with VaIN only (62.5%) were more likely to be negative for intraepithelial lesion or malignancy than patients with synchronous CIN (32.6%; p = 0.036). No statistically significant difference in HPV infection was noted between patients with VaIN and those with vaginitis (p = 0.439). The most prevalent HPV genotype in patients with VaIN or vaginitis was HPV16, whereas both HPV58 and HPV16 were the most common in patients with concurrent CIN.

**Conclusions:**

Attention should be paid to HPV16- and HPV58-positive patients with cytological abnormalities such as ASC-US and LSILs (especially with synchronous CIN) to avoid misdiagnosis or underdiagnosis and to facilitate early interventions for VaIN.

## Background

The incidence of vaginal intraepithelial neoplasia (VaIN) is difficult to accurately determine, given that VaIN is an asymptomatic disease. VaIN is rare, with an incidence of approximately 0.2–0.4 cases per 100,000 women, accounting for approximately 1% of all lesions in the epithelial layer of the lower genital tract [1–4]. VaIN is commonly regarded as a premalignant lesion that develop into an invasive squamous cell carcinoma in the vagina [5]. Early sexual initiation, having multiple sexual partners, tobacco consumption, and persistent human papillomavirus (HPV) infection are all reportedly associated with VaIN development [6]. Nonetheless, probable risk factors for VaIN progression have not been explicitly identified.

VaIN is classified similarly to cervical intraepithelial neoplasia (CIN), with HPV considered the major initiator. In particular, VaIN I is a benign form of HPV infection that typically resolves on its own, whereas VaIN II and III are considered precancerous lesions [7]. VaIN is often diagnosed via colposcopy-guided biopsy of areas that appear suspicious following abnormal cytology test results. As cytological screening becomes more widespread, the reported prevalence of this disease may also increase [8].

Various factors, such as age, HPV infection, cytology results, gravidity, parity, menopausal status, and number of male sexual partners, can increase an individual’s risk of VaIN; therefore, regular screening must be prioritized to avoid underdiagnosis. Owing to the rarity of VaIN, published data on the associated risk factors are limited. We have been surprised by the difficulty in reduce the underdiagnosis of VaIN. Given the limited data on risk factors, we conducted this retrospective analysis of VaIN in 62 patients with VaIN and 32 with vaginitis as a control group, with an emphasis on the importance of early detection of potential VaIN-related issues via vaginal examination and screening.

## Methods

### Data sources

A retrospective review was performed on women with histologically confirmed VaIN who were referred to a gynecological outpatient clinic at the Industrial Street Branch of Women’s and Children’s Central Hospital (Chengdu, China) from July 2020 to December 2021. All women diagnosed with VaIN or vaginitis via colposcopy-directed biopsy were included. VaIN and vaginitis were histologically diagnosed by two independent gynecologic pathologists. Women with synchronous lower genital malignancies, acute inflammation of the lower genital tract, or incomplete documentation were excluded from analysis. Those with vaginitis were included as a control group.

This study was conducted in accordance with the ethical principles embodied in the Declaration of Helsinki and was approved by the Ethics Committee of Chengdu Women’s and Children’s Central Hospital, reference number 2023(41). The requirement for the acquisition of informed consent from participants was waived by the Ethics Committee of Chengdu Women’s and Children’s Central Hospital owing to the retrospective nature of the data.

### Data collection

General clinical data, including age, menopausal status, gravidity, parity, number of sexual partners, HPV infection status, cytology results, and cervical epithelium condition were collected from hospital medical records.

### Statistical analyses

All statistical analyses were performed using GraphPad Prism software version 8.0 (GraphPad Software Inc., San Diego, CA, USA), with statistical significance set at p < 0.05. Categorical indicators were analyzed using the chi-square test or Fisher’s exact test, as appropriate.

## Results

### Prevalence and clinical data distribution

Table 1 details the clinical features of the study participants. A total of 62 patients with VaIN (mean age: 39.06 ± 11.66 years; range: 19–69 years) and 32 patients with vaginitis (mean age: 41.13 ± 13.43 years; range: 22–67 years) were included in this retrospective study. Age, menopausal status, gravidity, number of sexual partners, and the history of hysterectomy did not differ between patients with VaIN and those with vaginitis. With respect to parity, 83.9% of patients with VaIN and 53.1% of those with vaginitis reported having had no more than one pregnancy (p = 0.001). Synchronous CIN was histologically assessed and identified in 46 (74.2%) patients with VaIN and 7 (21.9%) with vaginitis, a statistically significant difference (p < 0.001). Among the 62 patients diagnosed with VaIN, 52, 8, and 2 patients had VaIN I, VaIN II, and VaIN III, respectively. Two patients were diagnosed with VaIN during post-hysterectomy follow-up for uterine leiomyoma and endometrial disease, one with VaIN I and the other with VaIN III. Interestingly, in both cases, cervical and vaginal wall biopsies were performed owing to abnormal cervical cancer screening results at a point before hysterectomy, which revealed no CIN lesions on the cervix.

**Table 1:** Patient characteristics

Characteristic	VaIN (*n* = 62)	Vaginitis (*n* = 32)	P-value
Mean age (years)	39.06 ± 11.66	41.13 ± 13.43	0.443
Postmenopausal			
Yes	13 (21.0%)	10 (31.3%)	0.533
No	49 (79.0%)	22 (68.8%)	
Gravidity			
≤ 1	22 (35.5%)	8 (25.0%)	0.302
≥ 2	40 (64.5%)	24 (75.0%)	
Parity			
≤ 1	52 (83.9%)	17 (53.1%)	0.001
≥ 2	10 (16.1%)	15 (46.9%)	
Sexual partners			
1	41 (66.1%)	17 (53.1%)	0.219
≥ 2	21 (33.9%)	15 (46.9%)	
Current smoker			
Yes	9 (14.5%)	6 (18.8%)	0.595
No	53 (85.5%)	26 (81.3%)	
Hysterectomy			
Yes	2 (3.2%)	0 (0.0%)	0.546
No	60 (96.8%)	32 (100.0%)	
Histopathology			
Concurrent CIN	46 (74.2%)	7 (21.9%)	< 0.001

Data are expressed as number of events (percentage) or mean ± standard deviation

VaIN, vaginal intraepithelial neoplasia; CIN, cervical intraepithelial neoplasia

Except for one patient with vulvar itching, almost all patients were asymptomatic but had abnormal cytology and/or HPV test results during regular screening for cervical cancer. Overall, 37 (59.7%) of patients with VaIN and 18 (56.3%) with vaginitis exhibited cytological abnormalities (p = 0.798). Among those, low-grade squamous intraepithelial lesions (LSILs) and atypical squamous cells of undetermined significance (ASC-US) were the most common in both groups, with no cases of atypical glandular cells on Pap smears (Table 2). Analysis of available cytology results revealed that 62.5% of patients with VaIN only and 32.6% of patients with both VaIN and CIN were negative for intraepithelial lesion or malignancy (NILM), a statistically significant difference (p = 0.036).

**Table 2:** Cytology test results for patients with VaIN and those with vaginitis

Cytology	VaIN (*n* = 62)	Vaginitis (*n* = 32)	*P*-value
NILM	25 (40.3%)	14 (43.8%)	0.798
ASC-US	22 (35.5%)	8 (25.0%)	
ASC-H	4 (6.5%)	3 (9.4%)	
LSILs	8 (12.9%)	6 (18.8%)	
HSILs	3 (4.8%)	1 (3.1%)	

Data are expressed as number of events (percentage)

VaIN, vaginal intraepithelial neoplasia; NILM, negative for intraepithelial lesion or malignancy; ASC-US, atypical squamous cells of undetermined significance; ASC-H, atypical squamous cells, HSIL cannot be excluded; LSILs, low-grade squamous intraepithelial lesions; HSILs, high-grade squamous intraepithelial lesions

Overall, 94.7% of the involved patients (60 patients with VaIN and 29 with vaginitis) developed an HPV infection. The HPV positivity rate was marginally higher in patients with VaIN (96.8%) than that in patients with vaginitis (90.6%); however, the difference did not reach statistical significance (p = 0.439). In patients diagnosed with HPV infection, the most prevalent type of HPV infection was a single, high-risk HPV infection (41.7% [*n* = 25] in patients with VaIN and 37.9% [*n* = 11] in patients with vaginitis). No cases of a single, low-risk HPV infection were observed among patients with vaginitis. Patients with vaginitis seemed more susceptible to multiple, high-risk HPV infections than patients with VaIN (p < 0.001) (Table 3). The most prevalent HPV genotypes were HPV16 in patients with VaIN and those with vaginitis, whereas both HPV58 and HPV16 were the most common in patients with concurrent CIN.

**Table 3:** HPV infection in patients with VaIN and in those with vaginitis

HPV infection status	VaIN (*n* = 60)	Vaginitis (n = 29)	*P*-value
Single, high-risk HPV infection	25 (41.7%)	11 (37.9%)	0.736
Multiple, high-risk HPV infections	12 (20.0%)	16 (55.2%)	< 0.001
Two HPV subtypes	9 (15.0%)	9 (31.0%)	
Three HPV subtypes	2 (3.3%)	7 (24.1%)	
Four HPV subtypes	1 (1.7%)	0 (0.0%)	
Multiple, high-risk HPV and low-risk HPV infections	12 (20.0%)	2 (6.9%)	0.200
Two HPV subtypes	8 (13.3%)	2 (6.9%)	
Three HPV subtypes	4 (6.7%)	0 (0.0%)	
Four HPV subtypes	0 (0.0%)	0 (0.0%)	
Single, low-risk HPV infection	3 (5.0%)	0 (0.0%)	0.548
Untyped HPV infection	8 (13.3%)	0 (0.0%)	0.049

Data are expressed as number of events (percentage)

VaIN, vaginal intraepithelial neoplasia; HPV, human papillomavirus

## Discussion

In this study, to increase sensitivity according to the literature, we used cytology combined with HPV screening to determine the need for biopsy [9]. Overall, 46 (74.2%) patients with VaIN had concurrent CIN. The most common cytological abnormalities were ASC-US (35.5%) and LSILs (12.9%). Additionally, 60 (96.8%) patients developed HPV infection. The most prevalent HPV genotypes were HPV16 in patients with VaIN and in those with vaginitis.

VaIN is often asymptomatic and not visible to the naked eye because exposing the natural vaginal folds and fornix during visual inspection is challenging; hence, the VaIN detection rate is relatively low. Patients often seek medical attention owing to abnormal cytology results and undergo colposcopy with multipoint biopsy to confirm any suspected VaIN lesions [10–12]. In the present retrospective study, we investigated various factors related to VaIN, such as age, HPV infection status, cytology results, gravidity, parity, menopausal status, and number of sexual partners, using data obtained from medical records to identify potential risk factors for VaIN and to provide evidence for early detection and intervention.

The age at which VaIN was diagnosed in this study was lower than that reported for previous studies [13–16]. Furthermore, in this study, the majority of patients were at the reproductive stage, with the remaining patients being perimenopausal. Menopause is a risk factor for VaIN, with postmenopausal women showing a 2.09-fold higher incidence than premenopausal women [17]. Postmenopausal women have decreased estrogen levels; thus, HPV easily traverses the thin vaginal epithelium. Factors such as having multiple sexual partners and multiparity are also associated with the occurrence and development of VaIN [18, 19]. However, the results of our study do not provide evidence to support the hypothesis that increased age, multiparity, and sexual promiscuity are associated with a higher risk of VaIN. Considering that our sample of patients with VaIN was relatively small compared to that of other studies, further research with larger samples is required to confirm these results.

HPV infects the host through metaplasia of squamous cells in the bruised or repaired vaginal and cervical mucosa. Different HPV subtypes can be detected locally in the vagina of patients with VaIN, and the prevalent HPV subtypes differ among regions [16]. In the present study, 96.8% of patients with VaIN developed an HPV infection, with high-risk HPV (particularly HPV16) being the most prevalent, which is consistent with the results of previous studies [5, 17, 19–22]. HPV58 and HPV16 were both common in patients with concurrent CIN, which is not completely consistent with the results of a previous report [14]. This may be owing to the prevalence of HPV58 in patients with CIN. In most instances, the differing distribution of HPV types between the vagina and cervix suggests that VaIN and CIN develop independently of each other [23]. The results indicated the close association of high-risk HPV infections with the occurrence of concurrent VaIN and CIN. Therefore, patients with a persistent HPV infection are recommended to undergo both cervical and vaginal examinations.

Because the cervix, vagina, and fornix are all located in the same environment, their cytology results could interfere with each other. Similar to those in previous studies, abnormal cytology results without clinical symptoms were the most common presentation in this study [24]. Our study revealed that patients who presented with VaIN alone had a notably higher percentage of NILM than those with concurrent CIN. In other words, patients with CIN were prone to having abnormal cytology results. With respect to cytological abnormalities, the proportions of ASC-US and LSILs were higher than those of other types, which is in line with the results of a previous study [15]. For patients with cytological abnormalities, particularly those with ASC-US and LSILs, a thorough examination of both the cervix and vagina is recommended. Available data support the use of abnormal cytology results as a marker of vaginal dysplasia.

The vaginal epithelium has the same origin as the cervical epithelium—namely, the common urogenital sinus. VaIN may be an extension of CIN in the vagina or a multicentric lesion predominantly occurring in the upper vagina that presents with concurrent CIN and vulvar intraepithelial neoplasia. Given that the cervix and vagina have the same histological source, the cervix adjacent to the vaginal fornix is the most common site for VaIN, which is why concurrent CIN is likely the most significant risk factor for VaIN. In this study, 74.2% of participants had both VaIN and CIN. As the coexistence of VaIN and CIN results in a lower rate of spontaneous regression than each lesion in isolation [25], factors related to concurrent CIN should be investigated. In clinical practice, the diagnosis of CIN is relatively straightforward, compared with that of VaIN, which is often missed owing to its lower incidence and visualization challenges.

HPV persistence is widely recognized as a significant risk factor for the development of primary and recurrent cervical dysplasia. Recent clinical research has revealed that patients with an HPV infection persisting for 12 months have a two-fold increase in recurrence compared to those with an infection persisting 6 months [26]. As one of the related risk factors of VaIN, the potential relationship between persistent HPV infection and VaIN recurrence is an avenue worth exploring. Thus, HPV persistence may predict the recurrence of VaIN.

Risk factors for VaIN progression have not been explicitly identified. A strength of this study is that the analysis was used to investigate the risk factors most likely to be associated with VaIN, to minimize the risk of misdiagnosis. This study has some limitations despite the informative data presented. First, its retrospective nature resulted in the risk of selection bias. Second, we did not analyze follow-up data on disease outcome, so there is a lack of factors related to disease outcome. Third, the study was conducted in a single institution; thus, the results may not be generalizable. Finally, the sample was not large enough for meaningful statistical analysis. For this point, we refer to previously published articles exploring the relationship between HPV and VaIN development [19]. Despite these limitations, cytology and HPV data were available for each study participant. A key strength of this study is its novelty, as this is the first study to taking CIN as a key point in exploring VaIN-related factors. Therefore, our results are still valuable in helping doctors during decision-making and in spurring on further research.

## Conclusions

Our results suggest that VaIN is associated with a high-risk HPV infection, abnormal cytology results, and concurrent cervical lesions. Further multicenter research with a larger sample is warranted to further study the clinical importance of age, multiparity, and sexual promiscuity in VaIN. Attention should be paid to HPV16- and HPV58-positive patients with cytological abnormalities such as ASC-US and LSILs (especially those with concurrent CIN) to avoid misdiagnosis or underdiagnosis and to facilitate early interventions for VaIN.

## Data Availability

The datasets used and/or analyzed during the current study are available from the corresponding author on reasonable request.

## List of abbreviations

ASC-H: Atypical squamous cells, HSIL cannot be excluded
ASC-US: Atypical squamous cells of undetermined significance
CIN: Cervical intraepithelial neoplasia
HPV: Human papillomavirus
HSILs: High-grade squamous intraepithelial lesions
LSILs: Low-grade squamous intraepithelial lesions
NILM: Negative for intraepithelial lesion or malignancy
VaIN: Vaginal intraepithelial neoplasia
